# Heat-induced stress modulates cell surface glycans and membrane lipids of coral symbionts

**DOI:** 10.1093/ismejo/wraf073

**Published:** 2025-04-18

**Authors:** Giada Tortorelli, Sabrina L Rosset, Clarisse E S Sullivan, Sarah Woo, Erika C Johnston, Nia Symone Walker, Joshua R Hancock, Carlo Caruso, Alyssa C Varela, Kira Hughes, Christian Martin, Robert A Quinn, Crawford Drury

**Affiliations:** Hawai‘i Institute of Marine Biology, University of Hawai‘i at Mānoa, Kāne‘ohe, HI 96744, United States; Hawai‘i Institute of Marine Biology, University of Hawai‘i at Mānoa, Kāne‘ohe, HI 96744, United States; Department of Biochemistry and Molecular Biology, Michigan State University, East Lansing, MI 48823, United States; Hawai‘i Institute of Marine Biology, University of Hawai‘i at Mānoa, Kāne‘ohe, HI 96744, United States; Hawai‘i Institute of Marine Biology, University of Hawai‘i at Mānoa, Kāne‘ohe, HI 96744, United States; Hawai‘i Institute of Marine Biology, University of Hawai‘i at Mānoa, Kāne‘ohe, HI 96744, United States; Hawai‘i Institute of Marine Biology, University of Hawai‘i at Mānoa, Kāne‘ohe, HI 96744, United States; Hawai‘i Institute of Marine Biology, University of Hawai‘i at Mānoa, Kāne‘ohe, HI 96744, United States; Hawai‘i Institute of Marine Biology, University of Hawai‘i at Mānoa, Kāne‘ohe, HI 96744, United States; Hawai‘i Institute of Marine Biology, University of Hawai‘i at Mānoa, Kāne‘ohe, HI 96744, United States; Hawai‘i Institute of Marine Biology, University of Hawai‘i at Mānoa, Kāne‘ohe, HI 96744, United States; Department of Biochemistry and Molecular Biology, Michigan State University, East Lansing, MI 48823, United States; Department of Biochemistry and Molecular Biology, Michigan State University, East Lansing, MI 48823, United States; Hawai‘i Institute of Marine Biology, University of Hawai‘i at Mānoa, Kāne‘ohe, HI 96744, United States

**Keywords:** Symbiodiniaceae, coral symbiosis, glycans, lipids, *Montipora* capitata, heat stress, oxidative stress, ROS, betaine lipids, metabolomics

## Abstract

The susceptibility of corals to environmental stress is determined by complex interactions between host genetic variation and the Symbiodiniaceae family community. We exposed genotypes of *Montipora capitata* hosting primarily *Cladocopium* or *Durusdinium* symbionts to ambient conditions and an 8-day heat stress. Symbionts’ cell surface glycan composition differed between genera and was significantly affected by temperature and oxidative stress. The metabolic profile of coral holobionts was primarily shaped by symbionts identity, but was also strongly responsive to oxidative stress. At peak temperature stress, betaine lipids in *Cladocopium* were remodeled to more closely resemble the abundance and saturation state of *Durusdinium* symbionts, which paralleled a larger metabolic shift in *Cladocopium*. Exploring how Symbiodiniaceae members regulate stress and host-symbiont affinity helps identify the traits contributing to coral resilience under climate change.

## Introduction

The symbiosis between dinoflagellate photosymbionts in the family Symbiodiniaceae and reef-building corals is the energetic and structural foundation of tropical coral reefs. The coral-algal symbiosis is evolutionarily ancient [1] and has persisted across significant environmental change [2], although the magnitude of contemporary climate variation may surpass the ability of this relationship to adjust or adapt. Rising sea surface temperature caused by anthropogenic climate change and high irradiance lead to oxidative stress *via* the accumulation of reactive oxygen species (ROS), which disrupts the mutualism between corals and Symbiodiniaceae symbionts and triggers bleaching [3, 4]. Coral bleaching has increased in frequency and severity since the 1980s and is expected to become an annual event on most reefs by mid-century [5].

The genetic variation in coral hosts and their symbiont community dictates holobiont susceptibility to environmental stress [6], consequently determining the present and future conditions of coral reefs under climate change. These associations hinge on molecular-level specificity and recognition between host and symbiont [7]. In higher animals, hosts detect microbes via pattern recognition receptors (PRRs) that bind to specific microbial-associated molecular patterns (MAMPs) and trigger a response [8]. Cnidarians possess PRRs like lectins [9–17], and symbiont cells of the family Symbiodiniaceae exude MAMPs on their surface as glycoconjugates [18–20], varying by species [21–25]. PRRs-MAMPs signaling is an active area of research in the Cnidaria-Symbiodiniaceae field [15–18, 22–29], and their compatibility may explain specificity between partners [8, 22–25]. Lectin-glycan research mainly focused on symbiosis in horizontally transmitting species, focusing less on vertically transmitting ones (maternally inherited symbionts) and how this system maintains and adapts under stress [7]. Understanding this process is crucial for coral reef persistence and applies to both transmission modes; though the molecular mechanism remains unclear, it could be key to mediating these interactions.

*Montipora capitata* is a broadcast spawner that vertically transmits symbionts *via* symbiont-provisioned eggs. In Kāneʻohe Bay, Hawaiʻi, it forms symbioses with either *Cladocopium* spp., *Durusdinium* spp., or a mix of both symbionts [30]. *Cladocopium* is the most diverse and widespread genus in the Symbiodiniaceae family, whereas *Durusdinium* includes thermotolerant species that resist disassociation from their host and can increase in abundance after heat stress [1, 31] and enhance the holobiont's thermotolerance [32–34]. Bleaching tolerance in *M. capitata* varies with its symbiont community. Corals with predominantly *Cladocopium* are typically more thermally sensitive than those dominated by *Durusdinium*, potentially buffering the effects of climate change [30, 35]. The susceptibility to bleaching is simultaneously manifested in the biochemistry of the coral animal and the algal symbiont, where betaine lipids, a class of glycerolipids characteristic of the plasma membrane of microalgae, are particularly strong indicators of bleaching sensitivity [36].

To explore the physiological, molecular, and metabolic traits of the *M. capitata* symbioses, we challenged *Cladocopium*- or *Durusdinium-*dominated coral genotypes from the same reef with a moderate 8-day heat stress. We compared their performance under heat to ambient conditions at three timepoints, assessing (i) photosynthetic efficiency, (ii) released ROS concentration, (iii) symbionts’ cell surface glycans, and (iv) the algal and coral metabolome.

## Materials and methods

### Experimental design and eDHW

**Figure 1:**
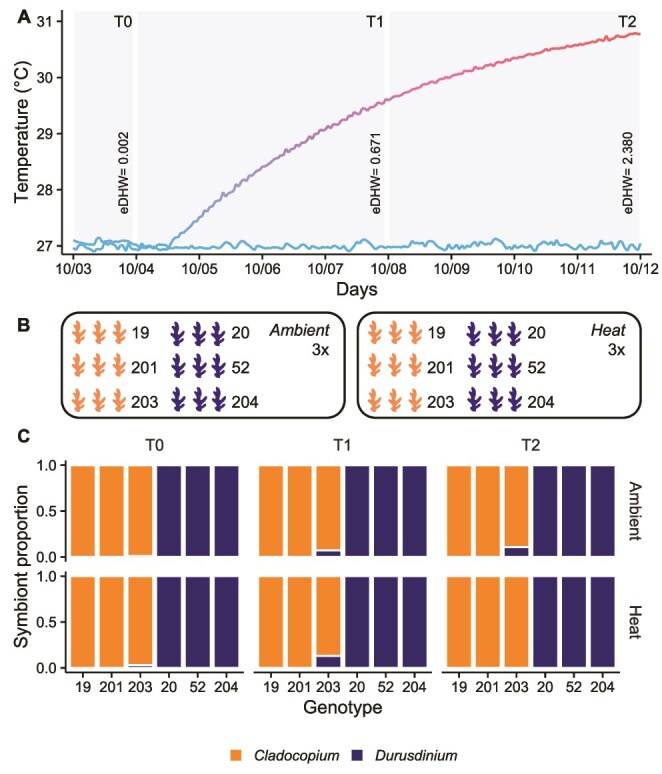
Experimental design. (A) Experimental timeline showing actual temperature records for ambient and heat treatments (T0 = day 0 corresponding to 0.002°C eDHW; T1 = day 4 corresponding to 0. 671°C eDHW; T2 = day 8 corresponding to 2.380°C eDHW). Lines represent temperatures recorded by the hour. (B) Three fragments per coral colony genotype (N = 108 total, of which genotypes 19, 201, 203 dominated by *Cladocopium* symbionts, and 20, 52, 204 dominated by *Durusdinium* symbionts) were kept in triplicate tanks at ambient or heated temperature. One fragment per coral genotype per tank was sampled at each timepoint. (C) Symbiodiniaceae ITS2 profiles in the six coral genotypes at each timepoint of the experiment in ambient and heat treatment (*Cladocopium*-dominated; *Durusdinium*-dominated).

We collected fragments from six known genotypes of *M. capitata* in Kāneʻohe Bay, Oʻahu, hosting *Cladocopium* (N = 3) or *Durusdinium* symbionts (N = 3) in June 2022 [6]. Fragments (N = 108) were transported to the Hawaiʻi Institute of Marine Biology, mounted on labeled plugs, and allowed to recover for three months in ambient flowthrough seawater tables under natural light. Following acclimatization, the corals were exposed to either ambient control temperature at 27°C (N = 3 tanks) or a heat profile gradually increasing to 31.5°C over 8 days (N = 3 tanks; Fig. 1A, B) at ambient light intensity (~ 42 μmol photons m^−2^ s^−1^, calculated as the average of the mid-point irradiance recorded for each day of the experiment). Each tank had three fragments per coral genotype. At each timepoint (T0 = day 2; T1 = day 6; T2 = day 8), we collected photosynthetic efficiency data for all remaining corals and randomly selected one coral fragment per tank per genotype for molecular analyses. The shape of the temperature profile results in timepoints for analyses, which do not fully incorporate the relationship between the magnitude of heat stress and physiological outcomes. Therefore, we also examined the relationship between physiological outcomes and experimental Degree Heating Weeks (eDHW [37]) in the heated treatment only. The heat treatment accumulated 0.0, 0.7, and 2.4°C-weeks at the three timepoints.

### Symbiodiniaceae members ITS2 analysis

To examine the genetic identity of algal symbionts within corals, each fragment was sampled with a 5 mm diameter dermal curette, promptly snap-frozen, and preserved at −80°C until subsequent analysis. Genomic DNA extraction was performed using the Zymo Quick DNA kit (Fisher Scientific, 50-444-148). The ribosomal internal transcribed spacer 2 (ITS2) region was targeted for amplification using the ITS-DINO and ITS2Rev2 primer pairs [38, 39], which were modified to include Illumina forward and reverse sequences. The polymerase chain reaction (PCR) for the ITS2 region followed the protocol: initial denaturation at 95°C for 3 min, 30 cycles of denaturation at 95°C for 30 sec, annealing at 55°C for 30 sec, extension at 72°C for 30 sec, and final extension at 72°C for 5 min. ITS2 amplicons were purified using Mag-Bind TotalPure NGS beads according to manufacturer protocol (OMEGA Bio-Tek, Norcross, GA, United States). A second round of PCR was conducted to attach unique dual Nextera XT primers (Set A and D) to individual samples, following the same thermal profile as the initial PCR but with 25 cycles. We used Hotstart 2x mastermix for both PCR rounds. Following purification with Mag-Bind TotalPure NGS beads and normalization based on determined concentrations (QuantIT kit, Qiagen, Hilden, Germany), the libraries were pooled for sequencing on the MiSeq System v3 (Illumina), generating 2x300 paired-end reads. Demultiplexed forward and reverse FASTq files were processed on SymPortal [40] to remove non-Symbiodiniaceae sequences and group Symbiodiniaceae sequences by genera.

### Photosynthetic performance

The performance of *in hospite* symbionts was assessed at each timepoint for all coral fragments with an Imaging Pulse-Amplitude Modulation chlorophyll fluorometer (IPAM M-series, Walz, Germany) using ImagingWin software (v2.56zg) [41, 42]. We measured the maximum quantum yield (*Fv/Fm*) of symbionts’ photosystem II using 2-h dark-adapted coral fragments [40]. Each coral was a biological replicate, with three-point areas of interest (AOIs) averaged to provide technical replication. Measurements were acquired using the following settings: measuring light intensity = 2, saturation pulse = 8, gain = 2, and damping = 2.

### Symbiodiniaceae cells isolation

At each timepoint, three coral fragments per genotype (one from each tank) per treatment were sampled and airbrushed in 0.2 μm filtered seawater (FSW) to isolate symbiotic algae. The resulting homogenate was dissociated using a 22G1 needle to obtain single cells, centrifuged at 1000 × g for 5 min to pellet the symbiont cells, and resuspended in FSW. This procedure was repeated three times. Algal density for each sample was determined by triplicate counts with an automated cell counter (Invitrogen, Countess 3 FL), and the cell suspension was then aliquoted for ROS and surface glycans assays.

### Symbiodiniaceae cells release of reactive oxygen species

Subsamples of the symbiont suspension (1 ml) were placed in a heat block matching control (27°C) or heat treatment (29.5°C for T1 and 31.5°C for T2) conditions for 3 h. Cells were pelleted by centrifugation at 3000 × g for 5 min, and 250 μl of supernatant was transferred into a black, clear bottom 96-well plate (CLS3603-48EA, Corning, NY, United States) in triplicates for ROS measurements. To measure the extracellular ROS that has leaked from the symbiont cells [43, 44], 0.5 μl of CellROX orange reagent probe (C10443, ThermoFisher Scientific, Waltham, MA, United States) was pipetted into each well at 5 μM final concentration. The mix was incubated at 37°C for 30 min in the dark, and the ROS signal was measured in a plate reader with 545-nm absorption and 565-nm emission. Measurements across plates were normalized to empty and blank measurements and cell numbers per milliliter, according to [43].

### Confocal microscopy of Symbiodiniaceae cell surface glycans

The symbiont cells isolated from three coral fragments per genotype per treatment were inspected at each timepoint for glycan analysis. The lectins ConA (Concavalin A, specific for D-mannose and D-glucose; C21401, ThermoFisher Scientific), LTL (*Lotus tetragonolobus* lectin, specific for L-fucose; L32480, ThermoFisher Scientific) and PNA (*Arachis hypogaea* lectin, specific for D-galactose; L21409, ThermoFisher Scientific), conjugated with the fluorescent molecular probes Alexa Fluor 488 (ConA, PNA) or FITC (LTL), were used to investigate binding affinity for the glycoconjugates present on the surface of symbiotic algae [23]. Symbiont cells (5 × 10^5^) in 1 ml subsamples were concentrated by centrifugation at 3000 × g for 5 min and fixed in 4°C cold 4% PFA/1x PBS for 3 h in darkness at 4°C. Following fixation, the samples were washed in 1x PBS and stored at 4°C. Each fixed symbiont cell sample was treated with each fluorescent lectin separately at a final concentration of 1 mg/ml and incubated in the dark for 1 h at room temperature. Subsequently, the samples were washed 3 times in 1x PBS, and the pelleted cells were resuspended in a final volume of 100 μl. Five μl aliquots from each sample were dispensed in four replicate wells of a Teflon-printed microscope slide, which was mounted with ProLong Gold antifade mounting medium (ThermoFisher Ccientific). Unstained controls were also prepared to profile the intrinsic autofluorescence of algal cells. Visualization of the samples was performed using a Zeiss LSM-710 confocal microscope with Zeiss Zen Imaging Software (Black edition). Signal acquisition from the fluorophores Alexa Fluor 488 and FITC, associated with the different lectins, was achieved by recording variable emission bandwidth (500–550 nm for AF 488 and FITC; 660–710 nm for chlorophyll autofluorescence). A 488 nm laser was employed to excite both the fluorophores and the dinoflagellate chlorophyll autofluorescence (laser intensity 15, gain 500). Imaging was conducted using a 63x magnification oil immersion objective, capturing three sections in each well and obtaining 12 observations per sample per lectin treatment. The microscopic analysis of samples visually examined whether host-derived membranes surrounded the algal cells, and we specifically analyzed symbiont cells that appeared symbiosome-free. Image processing was performed as described previously [23]. Briefly, acquired images were unmixed against negative controls to remove the signal caused by algal chlorophyll autofluorescence. An ImageJ macro was used to obtain mean fluorescence intensity values (MFIs) of the signal provided by the lectins binding to symbiont cell surface glycans. The macro splits each confocal image into two separate color channels: the “nucleus” (chlorophyll fluorescence) and the “membrane” (lectin probe fluorescence) channels. The symbiont cells (nucleus channel) are segmented and analyzed using thresholding and watershed algorithms, and the cell surface-associated fluorescence intensity (membrane channel) is measured by generating a ring mask around the cells in the nucleus channel. Thirty-two algal cells were randomly selected for each sample and lectin, yielding 96 symbiont cells analyzed for each coral genotype, treatment, timepoint, and lectin.

### Metabolomics

At each timepoint, three coral fragments per genotype per treatment were sampled with a 5 mm diameter dermal curette and transferred directly to 1.5 ml amber glass vials containing 500 μl of 70% HPLC-grade methanol. Samples were kept on ice for 30 min and stored at −80°C until shipping on dry ice to Michigan State University for untargeted metabolomics analysis. A Thermo Q-Exactive mass spectrometer coupled to a Vanquish Ultra High-Performance Liquid Chromatography (UHPLC) system was used to analyze methanolic extracts.

Following thorough vortexing of each sample vial, 50 μl of the methanol extract was added to a 96-well sample plate and diluted 1:1 in 50% methanol containing 2.5 mg/ml phenol red (phenolsulfonphthalein) as an injection standard. The mobile phase was 0.1% formic acid in HPLC-grade water (channel A) and acetonitrile (channel B). The stationary phase was a reverse phase column Waters Acquity (Wood Dale, IL, USA) UPLC BEH C-18 column, 2.1 mm × 100 mm. The chromatographic runs were 12 min-long with linear gradients as follows: 0–1 min 2% B, 1–8 min 2–100% B. The 100% B solution was held for 2 min followed by a switch to 2% B for an additional 2 min. The injection volume was 10 μl, the flow rate 0.40 ml/min and the column temperature 60°C. Full MS1 survey scans and MS2 mass spectra for five precursor ions per survey scan were collected using electrospray ionization in positive ionization mode with a scan range set from m/z 100 to 1500 for the full MS mode (min 1–10 of run). Solvent blank and quality control samples consisting of a sample pool were run every 10 samples. Raw files were converted to .mzXML format and processed with MZmine 3.9.0 software, GNPS, and SIRIUS [45–47].

Feature detection for MS1 and MS2 was performed for a centroid mass detector with a signal threshold of 5.0E4 and 1.0E4 counts respectively. Chromatogram builder was run considering a minimum height of 1.0E5 and a m/z tolerance of 10 ppm. Chromatograms were deconvoluted using the local minimum search algorithm with a peak duration range of 0.0 to 3.00 min and a baseline cut-off algorithm of 1.0E5. Isotopic peaks were grouped with a m/z tolerance of 0.02 Da and a retention time percentage of 0.05. Detected peaks were aligned through Join Aligner Module considering 0.02 Da and retention time tolerance of 0.02 min. The resulting peak list was gap-filled, considering an intensity tolerance value of 0.001, 0.02 Da, and retention time tolerance of 0.02 min. The data were converted to .mgf format and a feature quantification table was generated for Feature-Based Molecular Networking (FBMN) workflow on GNPS [47–49]. FBMN was performed with a parent and fragment mass ion tolerance of 0.02 Da, a cosine score of 0.65, and a minimum matched peaks minimum of 4, and these same parameters were used to obtain library matches (available at GNPS). *In silico* chemical classification of metabolite features was performed using CANOPUS in SIRIUS. Compound annotations were also determined by mzCloud (Thermofisher Scientific) spectral database search in Compound Discoverer. Compound annotations for the metabolite classes DGCC, PC, and fatty acylcarnitines were manually verified. Prior to statistical analysis, features with a mean signal intensity greater than three times higher in blanks compared to samples were removed. The remaining features were normalized to the summed signal intensity of each sample to calculate the relative abundances of each feature.

### Statistical analysis

All statistical analyses were conducted using R studio v. 4.1.2 [50]. Each genotype was classified by its dominant symbiont community using ITS2 data. Tank effect was assessed with generalized linear models (GLM, family = gaussian) for each response variable [outcome ~ tank * treatment]; variance associated with the tank factor was not significant for any dependent variables in our experiment, so we conducted downstream analysis excluding this factor. After preliminary analyses, genotypic effects were important for model fits, so we incorporated the genotype factor in various ways.

To assess photosynthetic efficiency and ROS release/cell, we used a generalized linear model (GLM) including treatment, sampling timepoint, host genotype, and symbiont genus (nested within host genotype) [outcome ~ treatment * timepoint * (symbiont/genotype)]. We also used GLM to assess the impact of accumulated heat stress (eDHW [37]), host genotype, and symbiont genus (nested within host genotype) on photosynthetic efficiency and ROS release/cell [outcome ~ eDHW * (symbiont/genotype)]. We used a linear mixed-effects model (LMEM) implemented in the *lmerTest* package [51] to assess the relationship between photosynthetic efficiency and ROS release/cell, including symbiont nested within host genotype as a random effect [*Fv/Fm* ~ ROS/cell + (1| symbiont/genotype)]. Model predictions were obtained by using the function “ggpredict” in the *ggeffects* package [52] [model, terms = eDHW, symbiont, genotype]. ROS values were log_2_-transformed for model prediction and visualization. For all models, we used ANOVA to evaluate the main effects and Tukey’s HSD test implemented in the *emmeans* package [53] for *post hoc* comparisons.

For the glycan analyses, we used PERMANOVA (999 permutations) and β-dispersion analysis [54, 55] in the *vegan* package [56] to analyze the MFI profiles of ConA, LTL, and PNA in the two symbiont types and the multivariate homogeneity for *Cladocopium* and *Durusdinium* symbionts at control conditions. We used a generalized linear mixed model (GLMM, family = gaussian) implemented in the *glmmTMB* package [57] to explore the impact of treatment, timepoint, glycan, and symbiont genus on MFI profiles, including host genotype as a random effect [MFI ~ treatment * timepoint * symbiont * glycan + (1|genotype)]. ANOVA was used to evaluate the main effects, and Tukey’s HSD test was used for *post hoc* comparisons. We used a similar generalized linear mixed model (family = gaussian) to evaluate the effects of glycan and ROS concentration on MFI profiles [MFI ~ glycan * ROS]. We evaluated the significance of these factors using the “lstrends” function in the *lsmeans* package [53], and determined a relationship was significant (*P* < 0.05) if the 95% CI of the slope did not overlap zero.

We used PERMANOVA (999 permutations) and β-dispersion analysis [54, 55] in the *vegan* package [56] to investigate the contribution of symbiont genus and temperature treatment to the biochemical response of these symbioses. The *caret* package [58] was used to implement a random forest classification of the symbiont genus using relative metabolite abundances. Variable importance for the random forest was calculated using the “varImp” function. We pre-filtered metabolites by comparing the relative abundance of each individual metabolite with ROS concentration of its respective sample using linear regression and removed metabolites with *P* > 0.05. We then used *caret* to implement a support vector machine (SVM) regression (polynomial kernel function) to associate metabolites with ROS concentration during heat stress. We calculated variable importance for the SVM regression by calculating the difference between the model’s best fit and the fit without each metabolite. The lipids identified in the random forest and SVM outputs were categorized by molecular class. The relative abundance (sum) of lipid species within each class was determined by integrating the spectral peak areas and calculating the percentage contribution of each peak area to the total. The unsaturation index (UI) for each lipid class was calculated as $\Big\{ UIx=\left[y\ \left(\% lipid\ y\ x\ total\ number\ of\ double\ bonds\ lipid\ y\right)\right]/100$, where *y* is every molecular lipid species belonging to the lipid class *x* [59]. We compared sum and UI across symbiont genera and treatments with GLMs (outcome ~ treatment * symbiont), used ANOVA to evaluate the main effects in the model, and Tukey’s pairwise tests for *post hoc* comparisons with the “contrast” function in the *emmeans* package [53]. We calculated pairwise Euclidean distances between samples using all metabolites and filtered the data to contrast ambient and high treatments for *Cladocopium* and *Durusdinium* symbionts separately. These distances represent the metabolomic shift of each symbiont genus from ambient to high treatments, which were compared between genera using a Wilcoxon test.

## Results

### Symbiodiniaceae members ITS2 analysis

Three coral genotypes were dominated by *Cladocopium,* and three genotypes were dominated by *Durusdinium* symbionts*,* reproducing the previous identification of these colonies from 2018 [30]. Sequencing yielded an average of 66 372 raw reads (range:12 289–631 176). Following quality filtering using SymPortal, the average number of reads was 41 008 (range: 2880–387 381). SymPortal identified 14 types of *Cladocopium* and 11 types of *Durusdinium*, which were condensed into nine type profiles (Supplementary Fig. 1). We refer to the two symbiont groups as *Cladocopium* and *Durusdinium* throughout the remainder of the text. The symbiont community composition remained stable within each genotype across the study (Fig. 1C and Supplementary Fig. 1). A single *Cladocopium*-dominated colony (#203) had a background population of *Durusdinium* cells (< 30%; Fig. 1C).

### Physiology

Treatment, timepoint, and symbiont significantly influenced *Fv/Fm* throughout the experiment (*P_GLM_* < 0.001), and there was a significant treatment-timepoint interaction (*P_GLM_* = 0.011; Supplementary Table 1; Supplementary Fig. 2). *Cladocopium*-dominated corals exhibited significantly higher photosynthetic efficiency than corals hosting *Durusdinium* throughout the experiment in both treatments (Tukey’s HSD, *P_C-D_* < 0.001). Due to our deliberately moderate heat stress, the heat treatment did not significantly impact *Fv/Fm* experiment-wide until the final timepoint (Tukey’s HSD, *P_T2.A-H_* < 0.05; Supplementary Fig. 2), when both symbiont genera experienced similar declines compared to control, leading to the significant main effect of treatment.

**Figure 2:**
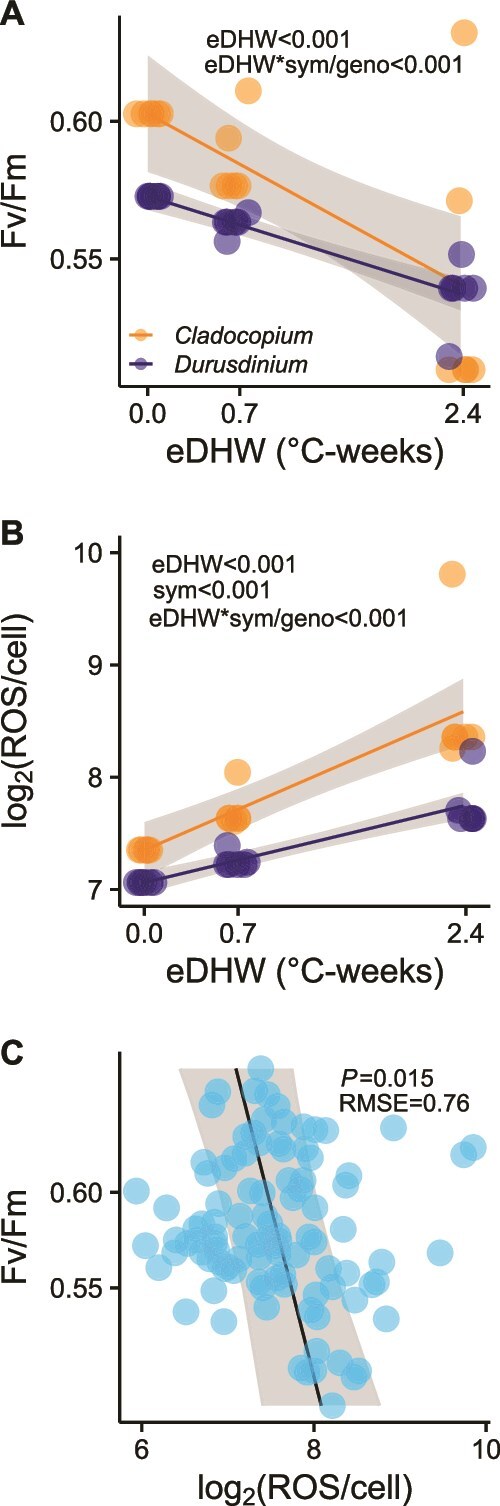
Symbiodiniaceae physiological analyses. (A) Model prediction of symbionts’ photosynthetic efficiency (*Fv/Fm*) and experimental degree heating weeks (eDHW; °C-weeks) at heat treatment. *Cladocopium*-dominated corals (N = 42); *Durusdinium*-dominated corals (N = 53). (B) Model prediction of ROS released by symbionts and experimental degree heating weeks (eDHW; °C-weeks) at heat treatment. ROS release is measured in fluorescence, normalized to algal cell numbers, and log_2_ transformed. *Cladocopium*-dominated corals (N = 23); *Durusdinium*-dominated corals (N = 26). (C) Correlation between symbionts’ photosynthetic efficiency and log_2_(ROS) /cell in all treatments and timepoints. Ambient treatment (N = 53), heat treatment (N = 48).

The use of discrete timepoints during the heat-treatment analysis provides limited information about the relationship between the magnitude of heat stress and physiological outcomes because of the shape of the temperature profiles, so we also examined the relationship between physiological outcomes and experimental Degree Heating Weeks (eDHW [37]) in the heat treatment only. At the three time points, the heat treatment accumulated 0.0, 0.7, and 2.4°C-weeks. There was a significant negative relationship between eDHW and photosynthetic efficiency (*P_GLM_* < 0.001; Fig. 2A; Supplementary Table 1). The influence of host genotype obscured an interaction between eDHW and symbiont community on *Fv/Fm* (*P_GLM_* = 0.55), but we found a significant 3-way interaction when including genotype as a nested variable for symbiont (*P_GLM_* < 0.001), suggesting substantial heterogeneity within symbiont genera due to the individual holobiont.

Timepoint and symbiont significantly influenced symbiont ROS release (*P_GLM_* < 0.001; Supplementary Table 2). There was no significant main effect of treatment (*P_GLM_* = 0.11), but there was a significant treatment-symbiont interaction (*P_GLM_* = 0.019), as well as a treatment-timepoint-symbiont-genotype interaction (*P_GLM_* < 0.001). The initial concentration of released ROS was similar between the two genera (Tukey’s HSD, *P_T0.C-D_* = 0.54) and remained relatively stable in samples that were not subjected to the heat treatment (Tukey’s HSD, *P_A.T2.C-D_* = 0.90; Supplementary Fig. 3). The interactive effects were driven by the increase in ROS release in two of three genotypes hosting *Cladocopium* members at the final timepoint (Tukey’s HSD, *P_H.T2.C-D_* < 0.001), resulting in significantly higher ROS in the heat than ambient conditions (Tukey’s HSD, *P_C.T2 A-H_* < 0.001; Supplementary Fig. 3). Conversely, the release of ROS by *Durusdinium* symbionts increased with rising temperatures but was not statistically different from ambient conditions (Tukey HSD, *P_D.T2.A-H_* = 0.06; Supplementary Fig. 3).

We explored the relationship between accumulated heat stress and ROS concentration in the heated treatment only. There was a significant positive relationship between ROS and eDHW (*P_GLM_* < 0.001), and *Cladocopium* symbionts released significantly more ROS than *Durusdinium* ones (*P_GLM_* < 0.001; Fig. 2B; Supplementary Table 2). We found a significant interaction between eDHW and symbiont community (*P_GLM_* < 0.001), and a 3-way interaction between eDHW, symbiont community, and host genotype on ROS release (*P_GLM_* < 0.001), suggesting increasingly severe oxidative stress in members of the *Cladocopium* genus.

We found a significant negative relationship between symbiont photosynthetic efficiency and ROS release (*P_LMEM_* = 0.015; RMSE = 0.76; Fig. 2C; Supplementary Table 3).

### Symbiodiniaceae cells surface glycans

**Figure 3:**
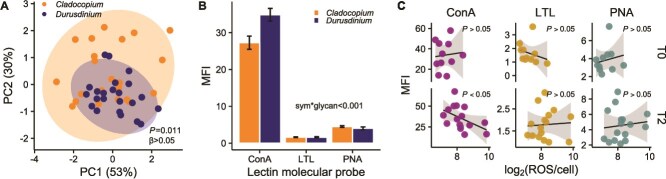
Symbiodiniaceae cell surface glycans analysis. (A) PCA of *Cladocopium* (isolated from coral samples N = 27) and *Durusdinium* symbionts (N = 27) surface glycans at ambient treatment; symbiont identity was compared using PERMANOVA, and variance was calculated using β-dispersion. (B) Mean fluorescence intensity (MFI) of the lectin molecular probes ConA (concanavalin A, specific for D-mannose and D-glucose), LTL (*Lotus tetragonolobus* lectin, specific for L-fucose), and PNA (*Arachis hypogaea* lectin, specific for D-galactose) on the surface of *Cladocopium* (N = 18 per glycan) and *Durusdinium* symbionts (N = 18 per glycan) isolated from coral samples throughout the experiment. (C) relationship between the MFI of the lectin molecular probes ConA, LTL, and PNA and ROS released by the symbionts into medium (measured in fluorescence and normalized to cell numbers; log_2_-transformed for visualization purposes) at the start of the experiment (T0, N = 18) and at the highest temperature stress (T2, N = 18).

The surface of *Cladocopium* and *Durusdinium* symbiont cells was populated by glycans with affinity to the lectin conjugates ConA (concanavalin A), LTL (*L. tetragonolobus* lectin), and PNA (*A. hypogaea* lectin)*.* The MFI profiles of the three lectins were significantly different between the cell surface of *Cladocopium* and *Durusdinium* symbionts at control conditions (*P_PERMANOVA_* = 0.008; Fig. 3; Supplementary Table 4; Supplementary Fig. 4) and varied based on coral host genotype (*P_PERMANOVA_* = 0.047). The variance of the MFI profiles of the three lectins in the two symbiont genera was not statistically different (β-dispersion, *P* = 0.06; Fig. 3A).

ConA, which specifically binds to terminal D-mannose and D-glucose residues, had the strongest intensity profile, followed by PNA (specific for D-galactose) and LTL (specific for L-fucose; Fig. 3B). These differences led to a significant effect of glycan type on MFI profiles (i.e. various glycans had significantly different MFIs experiment-wide, *P_GLMM_* < 0.001; Fig. 3B; Supplementary Table 4). Treatment (*P_GLMM_* = 0.029) and sampling timepoint (*P_GLMM_* < 0.001) had a significant effect on MFI profiles. There was a significant interaction between the symbiont genus and glycan type (*P_GLMM_* < 0.001; Fig. 3B), driven by less abundant D-mannose/D-glucose (corresponding to weaker ConA MFI signal) and slightly more abundant D-galactose (corresponding to stronger PNA MFI signal) in members of the *Cladocopium* than members of the *Durusdinium* genus (Tukey HSD, *P_ConA.C-D_* = 0.004; *P_PNA.C-D_* = 0.045), supporting different cell surfaces between genera described by the PERMANOVA. We found a significant three-way interaction between treatment, sampling timepoint, and symbiont genus (*P_GLMM_* = 0.002).

We explored the relationship between the MFI profiles of the three lectin conjugates and reactive oxygen release in the heated treatment. There was a significant interactive effect of glycan and ROS concentration on MFI profiles (*P_GLMM_* = 0.013; Supplementary Table 4). This relationship was driven by a significant negative association between ROS concentration and the lectin ConA (specific for D-mannose and D-glucose; slope = −0.028; *P_GLMM_* < 0.05; Fig. 3C). There was no significant relationship between these factors under ambient conditions or between ROS and the lectins PNA and LTL in any treatment or timepoint, suggesting that this pattern emerges during heat stress.

### Metabolomics

**Figure 4:**
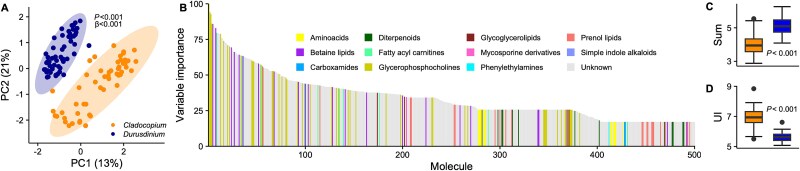
Biochemistry of symbiont diversity. (A) PCA of *Cladocopium* (N = 54) and *Durusdinium* (N = 54) corals metabolites; symbiont identity was compared using PERMANOVA, and variance was calculated using β-dispersion. (B) Top 500 metabolites with the strongest discriminatory power between symbionts genera, ranked by descending variable importance. Colors represent molecular families. (C) Abundance (sum) and (D) unsaturation index (UI) of lyso-DGCC lipids in *Cladocopium* and *Durusdinium* symbionts.

The liquid chromatography with tandem mass spectrometry (LC–MS/MS) metabolomic analysis identified 767 unique metabolite features. PERMANOVA resolved a significant differentiation of total metabolomic profiles between symbiont genera (*P_PERMANOVA_* < 0.001; Fig. 4A; Supplementary Table 5) and temperature treatments (*P_PERMANOVA_* < 0.001), with no significant interaction between symbiont and treatment (*P_PERMANOVA_* = 0.40) in the global metabolome. The biochemical distance within *Cladocopium*-dominated corals was significantly higher than *Durusdinium*-dominated corals (β-dispersion, *P* < 0.001; Fig. 4A). These analyses indicate symbiont genus was the main driver of biochemical variation; hence, we focused our analysis on the comparison between *Cladocopium* and *Durusdinium* metabolites.

A supervised random forest classification analysis identified metabolites driving the strongest discriminatory power between *Cladocopium* and *Durusdinium* symbionts (accuracy = 0.99, kappa = 0.98; Supplementary Table 5.1). *In silico* compound class prediction using Canopus identified 11 putative molecular families based on their spectral similarities to known compounds. Among these families, glycerophosphocholines (PC), betaine lipids (DGCC and DGTS), and fatty acylcarnitines emerged as particularly influential (Fig. 4B). We primarily detected lyso-forms of betaine lipids and PCs (*i.e.* having only one fatty-acyl chain), and explored the abundance (sum) and unsaturation index (UI) of these lipids. The lyso-diacylglycerylcarboxyhydroxymethylcholine (DGCC) lipids highlighted by the model displayed significant differences in abundance and saturation between symbiont types (*P_GLM_* < 0.001; Fig. 4C, D; Supplementary Fig. 5). Lyso-PC lipids with mono-acyl linkage were significantly different between *Cladocopium*- and *Durusdinium*-corals in saturation (*P_GLM_* < 0.001) but not in abundance (*P_GLM_* = 0.10; Supplementary Fig. 6A, B). Lyso-PC lipids with the mono-alkyl (ether) linkage (lyso-PAF) showed differences in both sum and unsaturation index (UI) between symbionts’ genera (*P_GLM_* < 0.001; Supplementary Fig. 6C, D).

**Figure 5:**
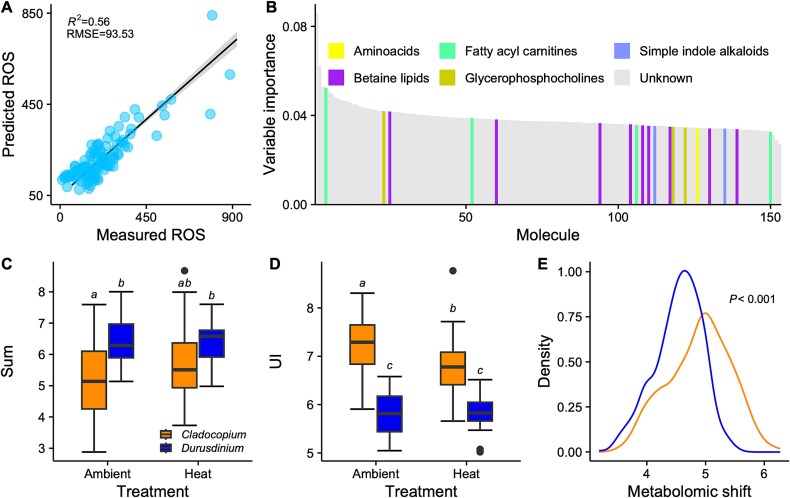
Metabolomics during stress. Pre-filtering of the metabolome dataset (N = 108) using linear models identified 153 metabolites associated with ROS concentration. (A) Prediction of the SVM poly regression model on the pre-filtered 153 metabolome (RMSE = 93.53) that explains 56% of the variation in measured ROS. (B) Top 153 metabolites associated with ROS concentration, resulting from the support vector machine analysis, and ranked by descending variable importance. Colors represent molecular families. (C) Overall lyso-DGCC abundance (sum) and (D) unsaturation index (UI) in *Cladocopium* and *Durusdinium* symbionts in ambient and heat treatments at the three timepoints. Letters represent *post hoc* significance values. (E) Metabolomic shift (pairwise Euclidean distance between samples for all metabolites) from ambient to high treatment for each symbiont genus.

Pre-filtering of the metabolome dataset using linear models identified 153 metabolites associated with ROS concentration (Supplementary Table 5.2). Using these variables, a support vector machine regression produced an ensemble model that explained 56% of the variation in measured ROS (*R*^2^ = 0.56, RMSE = 93.53; Fig. 5A), indicating a strong biochemical signature of oxidative stress. Many of the most important metabolites for predicting ROS abundance were betaine lipids (Fig. 5B). Symbiont genus was the main driver of the abundance of all identified lyso-DGCC lipids (*P_GLM_* < 0.001; Fig. 5C), with lower abundances of lyso-DGCC in *Cladocopium* than *Durusdinium* symbionts at ambient temperature (Tukey HSD, *P_A.C-D_* < 0.001). We found no significant effect of treatment (*P_GLM_* = 0.20) as all identified lyso-DGCC lipids in members of the *Durusdinium* genus remained constant (Tukey HSD, *P_A-H.D_* = 0.99), whereas in members of the *Cladocopium* genus they were slightly more abundant at heat compared to ambient conditions and equaled the abundance of *Durusdinium* at heat (Tukey HSD, *P_H.C-D_* = 0.33; Fig. 5C). The fatty acid saturation of all lyso-DGCC lipids identified was lower in *Cladocopium* than in *Durusdinium* symbionts (*P_GLM_* < 0.001; Fig. 5D). There was a treatment effect and a 2-way interaction between symbiont genus and treatment on the saturation of these lipids (*P_GLM_* < 0.05), where the UI of *Cladocopium* symbiont lipids decreased during the heat treatment compared to control (Tukey HSD, *P_A-H.C_* = 0.017; Fig. 5D) and *Durusdinium* symbiont lipids remained constant (Tukey HSD, *P_A-H.D_* = 0.99; Fig. 5D). A similar trend was found analyzing the subset of betaine lipids identified in the 153 metabolites associated with ROS concentration (Supplementary Fig. 7). Here, the betaine lipids in *Durusdinium* symbionts remained unaffected by exposure to heat stress (Tukey HSD, *P*_A-H.D_ > 0.05; Supplementary Fig. 7), while significantly decreasing in saturation in *Cladocopium* symbionts compared to ambient conditions (Tukey HSD, *P*_A-H.C_ = 0.008) and aligning with the lipid saturation in *Durusdinium* (Tukey HSD, *P*_H.C-D_ = 0.54; Supplementary Fig. 7B).

To ask if this pattern of convergence occurred more broadly across the metabolome, we used the abundance of all metabolites to calculate pairwise Euclidean distance between ambient and heated treatments for all *Cladocopium* and *Durusdinium* corals, separately. The biochemical distance between corals hosting *Cladocopium* was significantly higher than corals hosting *Durusdinium* (*P* < 0.001; Fig. 5E), suggesting a larger biochemical shift in *Cladocopium* members exposed to heat stress than *Durusdinium* members*.* Conversely, the distance between *Cladocopium* and *Durusdinium* symbionts in ambient and heat stress was not significantly different (*P* = 0.35), highlighting a targeted subset of metabolites responding to heat stress.

## Discussion

The persistence of coral reefs under climate change relies substantially on the identity and plasticity of the coral-Symbiodiniaceae mutualism. Here, we show that symbiont cell surface properties and biochemistry differ between members of *Cladocopium* and *Durusdinium* and change in response to heat stress. Combined host-symbiont effects strongly influenced the traits in our study, highlighting the individual holobiont as the physiological response unit rather than the symbiont genera in isolation. The collective impact of treatment and time highlights glycan responsiveness to heat stress. Higher temperatures altered the mannose and galactose on the symbiont cell surfaces and led to the convergence of betaine lipids biochemistry between thermosensitive *Cladocopium* and thermotolerant *Durusdinium* symbionts. The changes in symbiont cell surface glycoconjugates and membrane lipids were linked to heat-induced oxidative stress, suggesting shifts in the cellular dynamics of the symbiosis between *M. capitata* and its symbionts under stress.

The decline in photochemical efficiency, concomitant with increased ROS release by symbionts, suggests that a moderate temperature increase was sufficient to trigger thermal and oxidative stress in *M. capitata* (Fig. 2). Our results reveal a significant negative link between symbiont photochemical efficiency and ROS release (Fig. 2C), with ROS production arising from photosynthetic dysfunction in symbionts. As ROS accumulates, it overwhelms antioxidant defenses, damages photosynthetic membranes, and diffuses into the host tissue, ultimately leading to bleaching [4]. In this experiment, thermosensitive symbionts generally had higher photochemical efficiency and ROS release rate during heat stress than thermotolerant ones. Photosynthetic downregulation in *Durusdinium* symbionts confers higher thermal tolerance to corals, which experience milder oxidative stress as a result of lower photochemical efficiency compared to *Cladocopium* symbionts [30, 60–62]. Our results parallel the lower secreted ROS and improved thermal tolerance of heat-evolved *Cladocopium* strains from Australia compared to their wild-type, thermosensitive counterparts [43].

The differences in the MFI profile of ConA (specific for D-mannose and D-glucose) and PNA (specific for D-galactose) between *Cladocopium* and *Durusdinium* members align with the hypothesis that host lectins and symbiont glycans regulate partner specificity [63]. This suggests that host-symbiont combinations may be specific at or below the species level, potentially facilitating host genotype-specific associations. The timing of this molecular crosstalk is still unclear, as it may occur during initial contact (recognition [8]) or persist to stabilize the symbiosis and perhaps be involved in partners’ affinity. In a study that challenged *Acropora tenuis* larvae with homologous versus heterologous symbionts, the alteration of symbionts' cell surface glycans significantly impacted the number of symbionts in *A. tenuis* larvae but not the proportion of larvae infected, highlighting the role of cell surface glycans in post-uptake processes [29]. Based on this previous study and our results, the lectin-glycan mechanism seems to be maintained during symbiosis and supports coral-algal specificity.

At the highest point of heat stress, we recorded changes in the MFI profile of ConA on the symbionts’ cell surface, concurrent with increasing ROS release by thermally stressed symbionts (Fig. 3C). Mannose and glucose are among the most abundant saccharide moieties of Symbiodiniaceae cells surface glycoproteins [18, 23, 24], and their levels impact host-symbiont affinity. In a study on *Exaiptasia diaphana*, the homologous symbiont was rich in galactosylated glycans, and the experimental supplementation of galactose to the anemones enhanced the colonization efficiency of compatible symbionts. The incompatible algae, in contrast, were characterized by higher mannose glycans, and adding mannose to symbiont-free anemones reduced the colonization efficiency of the compatible algae [23, 24]. In another experiment, the heat stress of *Breviolum minutum* impacted the level of mannose on the surface of the alga and jeopardized their symbiotic affinity for the homologous cnidarian partner [25].

In our experiment, changes in the MFI profile of ConA suggest alterations of symbionts’ cell surface mannosylated and glucosylated glycans, which occurred at temperatures that triggered symbiosis disruption and were linked to oxidative stress. The connection between oxidative stress and glycans alteration has been defined “glyco-redox” effect, and it refers to the functional and structural changes of glycans driven by redox responses due to the generation of ROS [64]. Alterations in ROS levels can impact the composition of glycans associated with proteins and lipids *via* direct interaction of ROS and carbohydrate chains or through indirect modification of protein–protein interactions, molecular signaling, and genetic feedback [41]. In humans, ROS-mediated glycan changes are linked to inflammatory diseases and immune disorders [42, 65–67]. In the coral-algal symbiosis, the host mounts an immune response against incompatible intruders, allowing only compatible symbionts to bypass this immunologic reaction [8]. ROS-mediated glycan changes might disrupt compatibility, causing the coral to misidentify its symbiont as incompatible and destabilizing the mutualistic relationship.

The metabolic profile of *M. capitata* was strongly influenced by the symbiosis with either *Cladocopium* or *Durusdinium* symbionts (Fig. 4A). The main drivers of these differences were two lipid classes in their lyso-forms: betaine lipids and phosphatidylcholine (PC). Betaine lipids are a class of glycerolipids characteristic of the plasma membrane of microalgae. There are three types of betaine lipids, of which 1,2-diacylglyceryl-3-O-carboxy-(hydroxymethyl)-choline (DGCC) occurs in great amounts in the family Symbiodiniaceae, whereas Symbiodiniaceae also synthesizes DGTS, but at much lower abundance [68]. Phosphatidylcholine lipids are phospholipids with a choline headgroup and are significant components of cell membranes. We found two groups of phosphatidylcholine lipids: mono-acyl (lyso-PC) and mono-alkyl (lyso-PAF) lipids, which differ in having either an ester or an ether bond that attaches the fatty acid to the headgroup, respectively. Lyso-PAF is the precursor of the bioactive lipid PAF (Platelet Activating Factor), known to play a role in coral physiology and defense [69]. Only the symbiont synthesizes lyso-DGCC and lyso-DGTS, whereas both the host and the symbiont synthesize lyso-PC, and only the host synthesizes lyso-PAF [50, 70]. For these lipids, we measured abundance and saturation, factors that are linked to membrane fluidity and impacted by temperature fluctuations. A higher abundance of saturated lipids renders membranes more rigid, thus counteracting the effect of heat on the membrane structure. *M. capitata* with *Durusdinium* had a higher content of lyso-PAF and lyso-DGCC than with *Cladocopium* symbionts (Fig. 4C, D; Supplementary Fig. 6), whereas lyso-PC levels were similar (Supplementary Fig. 6). These metabolites have been identified before as strong predictors for *Cladocopium* and *Durusdinium* corals [36, 59]. The higher abundance of lyso-lipids in members of the *Durusdinium* genus may contribute to its thermal tolerance, but the mechanism remains unresolved.

Symbiodiniaceae-derived betaine lipids were top predictors of oxidative stress in the heat treatment. Lyso-DGCC levels increased in *Cladocopium* symbionts during stress, whereas *Durusdinium* remained unaltered (Fig. 5C, Supplementary Fig. 7A). In bacteria, lyso-lipid upregulation helps with protein stability during heat [71], and lipid unsaturation, linked to membrane fluidity, is crucial for thermal adaptation [72, 73]. In a previous study on *M.capitata* from Kāneʻohe Bay, Hawaiʻi, betaine lipids with higher degrees of saturation were significantly enriched in *Durusdinium* compared to *Cladocopium* members [36]. We observed a similar pattern: at the control temperature (27°C), lyso-DGCC in *Durusdinium* were more saturated than in *Cladocopium* symbionts (Fig. 5D). Heat stress did not impact members of the *Durusdinium* genus*.* Still, it altered the abundance and saturation of betaine lipids in *Cladocopium* members, making its profile resemble *Durusdinium* (Fig. 5C, D; Supplementary Fig. 7A, B). This trend was not unique to the DGCC betaine lipid form, as the lyso-DGTS type, though far less abundant in *M. capitata*, responded similarly (Supplementary Fig. 8). The remodeling of membrane lyso-lipids may help *Cladocopium* symbionts cope with stress, supporting the idea that lipid changes contribute to thermotolerance in symbiotic algae [36, 59]. Although the exact location of lyso-DGCC lipids in Symbiodiniaceae is unknown, betaine lipids are crucial components of algal biological membranes, and the adaptation and remodeling of DGCC abundance and saturation may assist with Symbiodiniaceae thermotolerance by contributing to the stability of extra-plastidial membranes [74].

Our results indicate that the physiological and metabolic profiles of *M. capitata* are strongly influenced by the symbiosis with *Cladocopium* or *Durusdinium* members. The different physiological responses to stress suggest that the thermotolerant *Durusdinium* maintains low photochemical efficiency and ROS release compared to the thermosensitive *Cladocopium*. During oxidative stress, symbiont surface mannose changes suggest ROS-induced glycan alterations may disrupt specific symbioses. Deviations from the stress-free symbionts’ cell surface glycan profile may play a role in the breakdown of specific partnerships and the plasticity of symbiosis under stress. However, our study focused on a few main glycan components of glycoconjugates, whereas further research should expand the analysis to a broader array of saccharides, symbiont species, and mixed symbiont communities cohabiting in the same coral. Although the analyzed symbiont cells visually seemed to be symbiosome-free, greater emphasis should be placed on labeling and identifying symbiosomes to ensure that lectin probes successfully bind to the glycans on the symbionts' membranes. We also documented a strong, lipid-mediated distinction between *Cladocopium* and *Durusdinium*-dominated corals at stress-free conditions. Under stress, *Cladocopium* symbionts remodeled their betaine lipids to resemble those of *Durusdinium* symbionts, which seems to be a strategy that Symbiodiniaceae members may rely on to cope with stress.

Different symbiont species can significantly buffer the effects of climate change. However, a critical gap remains in our understanding of the cellular mechanisms that support the recognition and maintenance of coral-symbiont mutualism. This study advances our knowledge of the cnidarian-Symbiodiniaceae symbiosis and sheds light on the cellular, physiological, and metabolic responses–and their interactions–in a photosynthetic organism during heat stress.

## Supplementary Material

Tortorelli_et_al_2024_Supplementary_figures_wraf073

Supplementary_Information_3_wraf073

## Data Availability

All data are available in the supplementary materials and at github.com/GiadaTortorelli/M.capitata_symbionts_glycans. Metabolomics data are available at GNPS. The massive repository accession number is MSV000095167.
