# Supplementary material for: Heat-induced stress modulates cell surface glycans and membrane lipids of coral symbionts
Source: ISME J. 2025 Apr 18;19(1):wraf073. doi: 10.1093/ismejo/wraf073 (PMC12077390; doi:10.1093/ismejo/wraf073)
Supplement: Tortorelli_et_al_2024_Supplementary_figures_wraf073 [file tortorelli_et_al_2024_supplementary_figures_wraf073.pdf]

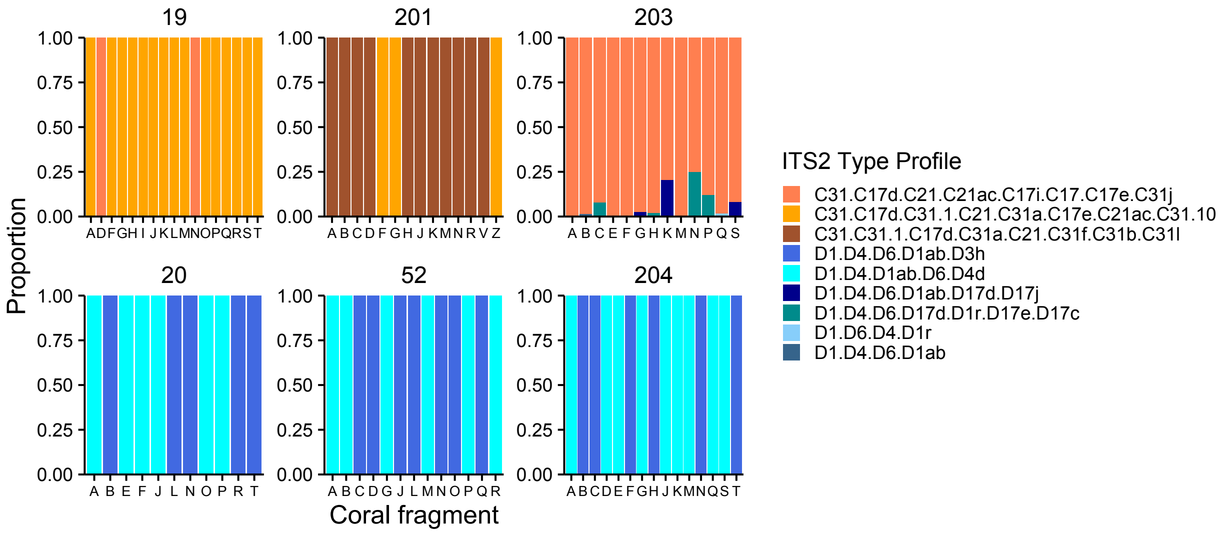

Supplementary Fig. 1. Symbiont identity. Symbiodiniaceae ITS2 type profiles for each coral fragment used in the study. Random letters are assigned to each coral fragment. Facets represent coral colony genotypes.

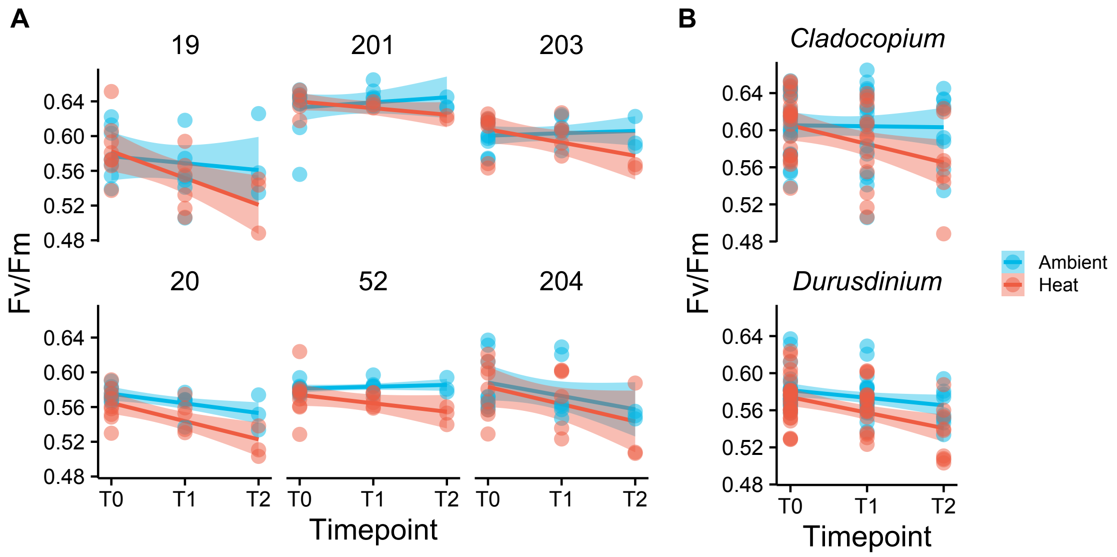

Supplementary Fig. 2. Symbiont physiology. A Photosynthetic efficiency (*Fv/Fm*) of symbionts in six coral genotypes at three timepoints at ambient (blue) and heat (red) treatments. Odd colony numbers represent *Cladocopium*-dominated corals, while even colony numbers represent *Durusdinium*-dominated corals. **B** Photosynthetic efficiency (*Fv/Fm*) of *Cladocopium*- and *Durusdinium*-dominated corals at three timepoints at ambient (blue) and heat (red) treatments.

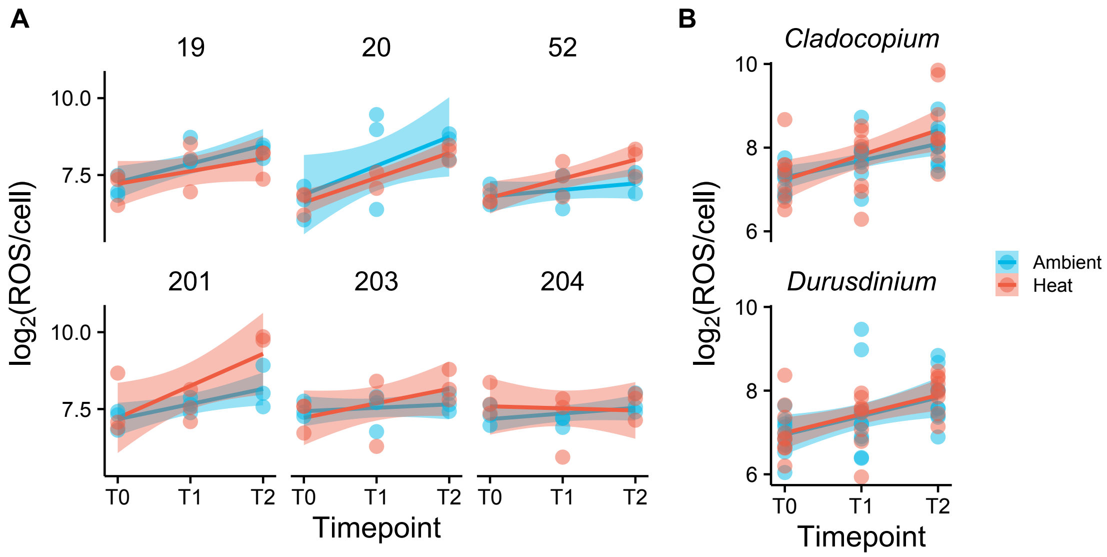

Supplementary Fig. 3. Symbiont physiology. A ROS released by symbionts isolated from six coral colony genotypes at three timepoints in ambient (blue) and heat (red) treatments. ROS release is measured in fluorescence, normalized to algal cell numbers, and $\log_2$ transformed. Odd colony numbers represent *Cladocopium*-dominated corals, while even colony numbers represent *Durusdinium*-dominated corals. **B** ROS released by *Cladocopium* and *Durusdinium* at three timepoints at ambient (blue) and heat (red) treatments.

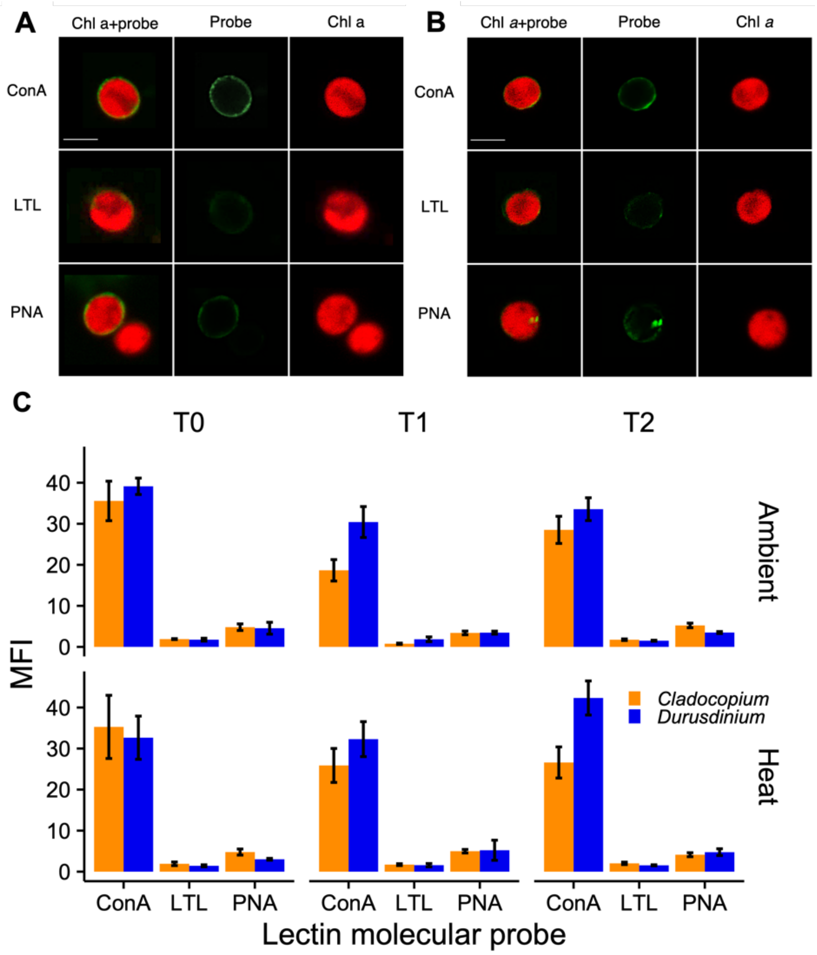

Supplementary Fig. 4. Symbiodiniaceae cell surface glycans confocal analysis.

Representative cells of **A** *Cladocopium* and **B** *Durusdinium* symbionts labeled with the lectin probes (green) ConA (concavalin A, specific for D-mannose and D-glucose), LTL (lectin from *Lotus tetragonolobus*, specific for L-fucose), PNA (lectin from *Arachis hypogaea*, specific for D-galactose) and imaged using confocal microscopy. Scale = 10 µm. **C** Mean fluorescence intensity (MFI) of ConA, LTL, and PNA on the surface of *Cladocopium* (orange) and *Durusdinium* (blue) symbionts isolated from coral samples across temperature treatments and timepoints. Ambient treatment: *Cladocopium* N = 27, *Durusdinium* N = 27; heat treatment: *Cladocopium* N = 27, *Durusdinium* N = 27.

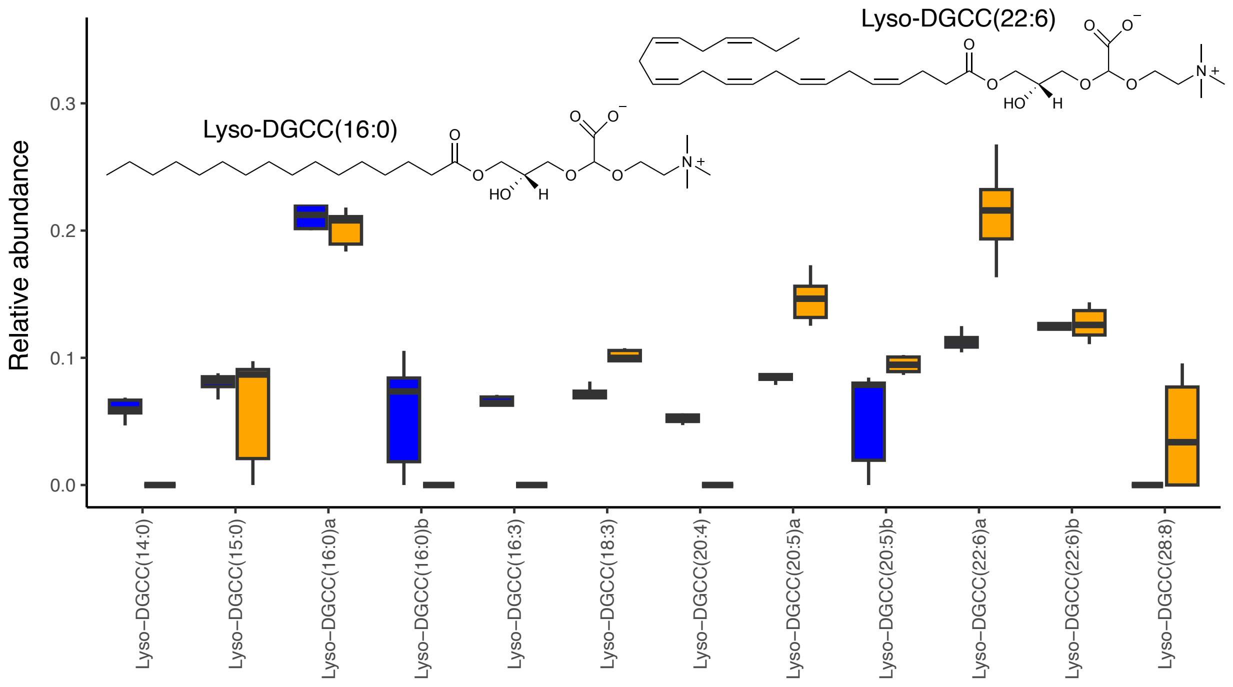

Supplementary Fig. 5. Lyso-DGCC lipids. A Relative composition of lyso-DGCC lipids in *Cladocopium* (orange) and *Durusdinium* (blue) corals.

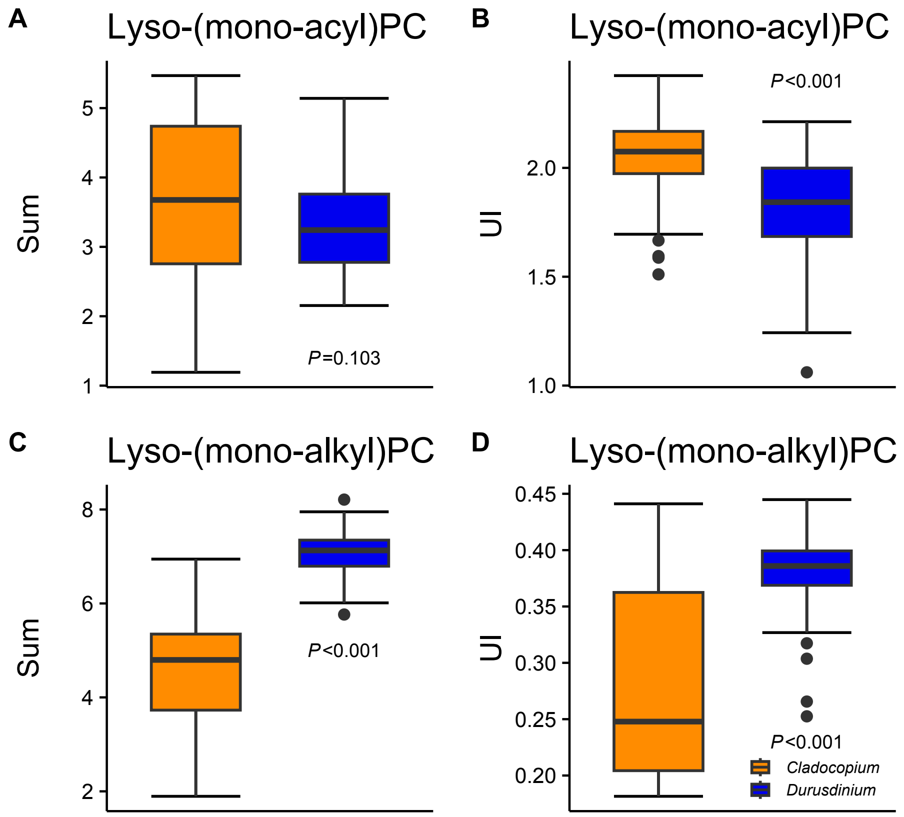

Supplementary Fig. 6. Lyso-lipids biochemistry of symbiont diversity. A Lyso-(mono-acyl)PC lipids abundance (sum) and **B** unsaturation index (UI) in *Cladocopium* (orange) and *Durusdinium* (blue) corals. **C** Lyso-(mono-alkyl)PC lipids abundance (sum) and **D** unsaturation index (UI).

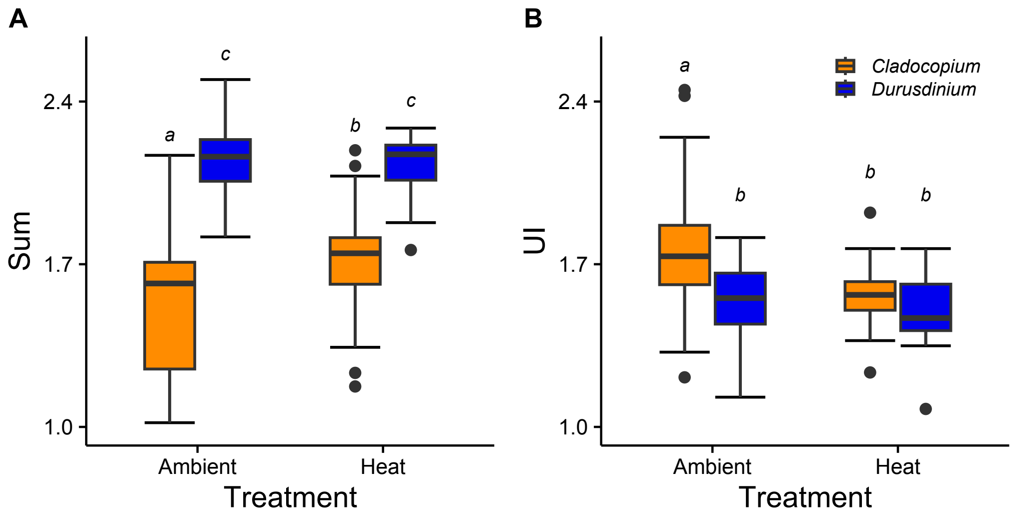

Supplementary Fig. 7. Lyso-DGCC lipids during stress. A Abundance (sum) and **B** unsaturation index (UI) of lyso-DGCC lipids in the top 153 metabolites associated with ROS concentration in *Cladocopium* (orange) and *Durusdinium* (blue) symbionts at ambient and heat treatments at the three timepoints. Letters represent *post hoc* significance values.

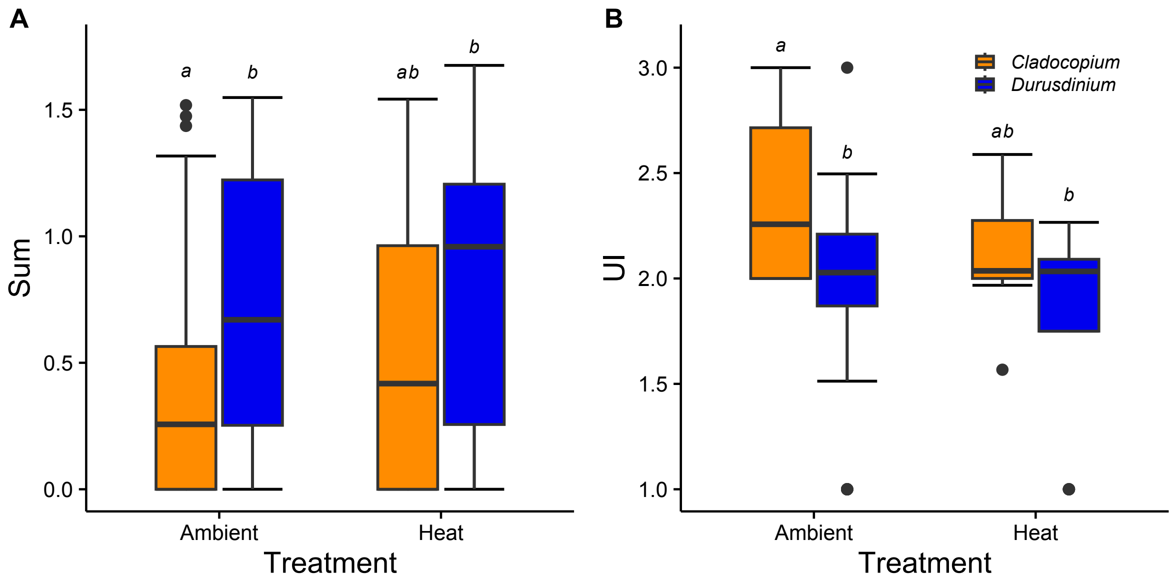

Supplementary Fig. 8. Lyso-DGTS lipids. A Overall lyso-DGTS lipids abundance (sum) and **B** unsaturation index (UI) in *Cladocopium* (orange) and *Durusdinium* (blue) in ambient and heat treatments at the three timepoints. Letters represent *post hoc* significance values.
